# Supplementary material for: Inter-Fork Strand Annealing causes genomic deletions during the termination of DNA replication
Source: eLife. 2017 Jun 6;6:e25490. doi: 10.7554/eLife.25490 (PMC5461108; doi:10.7554/eLife.25490)
Supplement: Figure 1—source data 1. — DOI: http://dx.doi.org/10.7554/eLife.25490.003 [file elife-25490-fig1-data1.docx]

**Figure 1 – Source Data 1.** Effect of inter-repeat distance on the frequency of *RTS1*-AO-induced direct repeat recombination.

| **Genotype and strain number** | ***RTS1***  **orientation** | **Extra DNA spacer between *ade6-M375* and *his3^+^*** | **Extra DNA spacer between *ade6-L469* and *his3*** | **Number of colonies analysed** | **Ade^+^ His^+^**  **recombinant**  **frequency (x 10^-4^)^a^** | | **Ade^+^ His^-^**  **recombinant**  **frequency (x 10^-4^)^a^** | |
| --- | --- | --- | --- | --- | --- | --- | --- | --- |
|  |  |  |  |  | **Mean** | ***P***  **value^b^** | **Mean** | ***P***  **value^b^** |
| wild-type MCW4712 | IO | - | - | 33 | 1.29  (+/- 0.40) | - | 3.23  (+/- 0.78) | - |
| wild-type MCW4713 | AO | - | - | 26 | 140.7  (+/- 39.3) | <0.001^c^ | 104.2  (+/- 29.0) | <0.001^c^ |
| wild-type MCW7020 | AO | 0.5 kb | - | 15 | 161.2  (+/- 71.1) | 0.24^d^ | 188.4  (+/- 57.2) | <0.001^d^ |
| wild-type MCW7021 | AO | 1.0 kb | - | 14 | 172.5  (+/- 52.9) | 0.08 ^d^ | 187.0  (+/- 54.1) | <0.001^d^ |
| wild-type MCW7022 | AO | 2.0 kb | - | 12 | 167.8  (+/- 25.4) | 0.05 ^d^ | 100.4  (+/- 23.5) | 0.67 ^d^ |
| wild-type MCW7749 | AO | 4.0 kb | - | 16 | 32.6  (+/- 8.1) | <0.001^d^ | 27.4  (+/- 8.8) | <0.001^d^ |
| wild-type MCW7748 | AO | 8.0 kb | - | 15 | 10.8  (+/- 3.8) | <0.001^d^ | 12.9  (+/- 2.3) | <0.001^d^ |
| wild-type MCW8019 | AO | - | 1.0 kb | 20 | 163.5  (+/- 76.7) | 0.58 ^d^ | 297.3  (+/- 150.4) | <0.001^d^ |
| wild-type MCW8020 | AO | - | 2.0 kb | 49 | 188.9  (+/- 54.8) | 0.01 ^d^ | 434.6  (+/- 148.0) | <0.001^d^ |
| wild-type MCW8021 | AO | - | 3.0 kb | 18 | 171.4  (+/- 46.2) | 0.02 ^d^ | 634.6  (+/- 191.3) | <0.001^d^ |
| wild-type MCW8022 | AO | - | 4.0 kb | 14 | 237.8  (+/- 61.6) | <0.001^d^ | 707.7  (+/- 230.0) | <0.001^d^ |
| wild-type MCW8023 | AO | - | 5.0 kb | 70 | 228.9  (+/- 140.2) | <0.001^d^ | 1103.0  (+/- 484.0) | <0.001^d^ |
| wild-type MCW8362 | IO | - | 5.0 kb | 19 | 0.82  (+/- 0.23) | 0.003^c^ | 3.55  (+/- 1.04) | 0.32^c^ |

^a^ The values in parentheses are the standard deviations about the mean.

^b^ *p* values are calculated by a two-tailed Mann-Whitney U test comparing the mean values as indicated.

^c^ Compared to the equivalent mean recombinant frequency for wild-type *RTS1-IO* (MCW4712).

^d^ Compared to the equivalent mean recombinant frequency for wild-type *RTS1-AO* with no extra DNA spacer (MCW4713).
