# Supplementary material for: Inter-Fork Strand Annealing causes genomic deletions during the termination of DNA replication
Source: eLife. 2017 Jun 6;6:e25490. doi: 10.7554/eLife.25490 (PMC5461108; doi:10.7554/eLife.25490)
Supplement: Figure 2—source data 1. — DOI: http://dx.doi.org/10.7554/eLife.25490.005 [file elife-25490-fig2-data1.docx]

**Figure 2 – Source Data 1.** Frequency of *RTS1*-AO-induced direct repeat recombination in wild-type, *rad51*∆ and *rad51*∆ *rad52*∆ strains with an extra 5 kb DNA spacer between the repeats.

| **Genotype and strain number** | ***RTS1***  **orientation** | **Extra DNA spacer between *ade6-L469* and *his3*** | **Number of colonies analysed** | **Ade^+^ His^+^**  **recombinant**  **frequency (x 10^-4^)^a^** | | **Ade^+^ His^-^**  **recombinant**  **frequency (x 10^-4^)^a^** | |
| --- | --- | --- | --- | --- | --- | --- | --- |
|  |  |  |  | **Mean** | ***P***  **value^b^** | **Mean** | ***P***  **value^b^** |
| wild-type MCW8023 | AO | 5.0 kb | 70 | 228.9  (+/- 140.2) | - | 1103.0  (+/- 484.0) | - |
| *rad51*∆ MCW8136 | AO | 5.0 kb | 29 | 17.2  (+/- 13.8) | <0.001^c^ | 1020.0  (+/- 276.2) | 0.76^c^ |
| *rad51*∆ *rad52*∆ MCW8138 | AO | 5.0 kb | 16 | 0.00  (+/- 0.00) | <0.001^c^ | 11.61  (+/- 9.35) | <0.001^c^ |

^a^ The values in parentheses are the standard deviations about the mean.

^b^ *p* values are calculated by a two-tailed Mann-Whitney U test comparing the mean values as indicated.

^c^ Compared to the equivalent mean recombinant frequency for wild-type *RTS1-AO* with 5 kb DNA spacer (MCW8023).
