# Supplementary material for: Inter-Fork Strand Annealing causes genomic deletions during the termination of DNA replication
Source: eLife. 2017 Jun 6;6:e25490. doi: 10.7554/eLife.25490 (PMC5461108; doi:10.7554/eLife.25490)
Supplement: Figure 3—source data 1. — DOI: http://dx.doi.org/10.7554/eLife.25490.007 [file elife-25490-fig3-data1.docx]

**Figure 3 – Source Data 1.** Effect of delaying fork convergence on the frequency of *RTS1*-AO-induced direct repeat recombination with and without an extra 5 kb DNA spacer between the repeats.

| **Genotype and strain number** | ***RTS1***  **orientation** | **Extra DNA spacer between *ade6-L469* and *his3*** | **Number of colonies analysed** | **Ade^+^ His^+^**  **recombinant**  **frequency (x 10^-4^)^a^** | | **Ade^+^ His^-^**  **recombinant**  **frequency (x 10^-4^)^a^** | |
| --- | --- | --- | --- | --- | --- | --- | --- |
|  |  |  |  | **Mean** | ***P***  **value^b^** | **Mean** | ***P***  **value^b^** |
| wild-type MCW4712 | IO | - | 33 | 1.29  (+/- 0.40) | - | 3.23  (+/- 0.78) | - |
| wild-type MCW4713 | AO | - | 26 | 140.7  (+/- 39.3) | <0.001^c^ | 104.2  (+/- 29.0) | <0.001^c^ |
| wild-type MCW8362 | IO | 5.0 kb | 19 | 0.82  (+/- 0.23) | 0.003^c^ | 3.55  (+/- 1.04) | 0.32^c^ |
| wild-type MCW8023 | AO | 5.0 kb | 70 | 228.9  (+/- 140.2) | <0.001^d^ | 1103.0  (+/- 484.0) | <0.001^d^ |
| *ori1253*∆ MCW6778^f^ | AO | - | 16 | 339.9  (+/- 83.6) | <0.001^d^ | 221.0  (+/- 57.2) | <0.001^d^ |
| *ori1253*∆ MCW8134 | AO | 5.0 kb | 39 | 298.4  (+/- 76.2) | <0.001^e^ | 478.2  (+/- 189.9) | <0.001^e^ |
| *rad51*∆ *ori1253*∆ MCW8456 | AO | 5.0 kb | 24 | 19.7  (+/- 36.8) | 0.19 ^g^ | 1004.5  (+/- 474.0) | 0.38 ^g^ |
| wild-type + *Ter2/3* MCW7290 | IO | - | 16 | 0.95  (+/- 0.30) | 0.006^c^ | 3.25  (+/- 0.93) | 0.72^c^ |
| wild-type + *Ter2/3* MCW7292 | AO | - | 15 | 199.1  (+/- 62.0) | <0.001^d^ | 194.6  (+/- 76.6) | <0.001^d^ |
| wild-type + *Ter2/3* MCW8592 | AO | 5.0 kb | 17 | 320.9  (+/- 77.1) | <0.001^e^ | 695.2  (+/- 166.2) | <0.001^e^ |

^a^ The values in parentheses are the standard deviations about the mean.

^b^ *p* values are calculated by a two-tailed Mann-Whitney U test comparing the mean values as indicated.

^c^ Compared to the equivalent mean recombinant frequency for wild-type *RTS1-IO* (MCW4712).

^d^ Compared to the equivalent mean recombinant frequency for wild-type *RTS1-AO* with no extra DNA spacer (MCW4713).

^e^ Compared to the equivalent mean recombinant frequency for wild-type *RTS1-AO* with 5 kb DNA spacer (MCW8023).

^f^ Data from Ref. (Nguyen et al., 2015).

^g^ Compared to the equivalent mean recombinant frequency for *rad51*∆ *RTS1-AO* with 5 kb spacer (MCW8136) (see Figure 2 – source data 1).
