# Supplementary material for: Inter-Fork Strand Annealing causes genomic deletions during the termination of DNA replication
Source: eLife. 2017 Jun 6;6:e25490. doi: 10.7554/eLife.25490 (PMC5461108; doi:10.7554/eLife.25490)
Supplement: Figure 4—source data 1. — DOI: http://dx.doi.org/10.7554/eLife.25490.010 [file elife-25490-fig4-data1.docx]

**Figure 4 – Source Data 1.** Frequency of *RTS1*-AO-induced direct repeat recombination in wild-type, *exo1*∆, *mus81*∆ and *fml1*∆ strains with and without an extra DNA spacer between the repeats.

| **Genotype and strain number** | ***RTS1***  **orientation** | **Extra DNA spacer between *ade6-L469* and *his3*** | **Number of colonies analysed** | **Ade^+^ His^+^**  **recombinant**  **frequency (x 10^-4^)^a^** | | **Ade^+^ His^-^**  **recombinant**  **frequency (x 10^-4^)^a^** | |
| --- | --- | --- | --- | --- | --- | --- | --- |
|  |  |  |  | **Mean** | ***P***  **value^b^** | **Mean** | ***P***  **value^b^** |
| wild-type MCW4713 | AO | - | 26 | 140.7  (+/- 39.3) | - | 104.2  (+/- 29.0) | - |
| wild-type MCW8020 | AO | 2.0 kb | 49 | 188.9  (+/- 54.8) | 0.01 ^c^ | 434.6  (+/- 148.0) | <0.001^c^ |
| wild-type MCW8023 | AO | 5.0 kb | 70 | 228.9  (+/- 140.2) | <0.001^c^ | 1103.0  (+/- 484.0) | <0.001^c^ |
| *exo1*∆ MCW1742^f^ | AO | - | 21 | 57.1  (+/- 11.6) | <0.001 ^c^ | 51.8  (+/- 23.3) | <0.001 ^c^ |
| *exo1*∆ MCW8589 | AO | 5.0 kb | 14 | 106.4  (+/- 39.1) | <0.001^d^ | 263.8  (+/- 114.6) | <0.001^d^ |
| *mus81*∆ MCW1452 | AO | - | 23 | 132.5  (+/- 42.3) | 0.30 ^c^ | 149.2  (+/- 77.8) | 0.013 ^c^ |
| *mus81*∆ MCW8434 | AO | 2.0 kb | 31 | 108.6  (+/- 43.9) | <0.001^e^ | 160.1  (+/- 64.4) | <0.001^e^ |
| *mus81*∆ MCW8402 | AO | 5.0 kb | 30 | 187.6  (+/- 98.0) | 0.06 ^d^ | 517.5  (+/- 155.9) | <0.001^d^ |
| *fml11*∆ MCW3061 | AO | - | 22 | 17.2  (+/- 8.9) | <0.001 ^c^ | 108.3  (+/- 53.5) | 0.65 ^c^ |
| *fml11*∆ MCW8300 | AO | 2.0 kb | 18 | 17.0  (+/- 38.5) | <0.001^e^ | 1186.9  (+/- 405.0) | <0.001^e^ |

^a^ The values in parentheses are the standard deviations about the mean.

^b^ *p* values are calculated by a two-tailed Mann-Whitney U test comparing the mean values as indicated.

^c^ Compared to the equivalent mean recombinant frequency for wild-type *RTS1-AO* with no extra DNA spacer (MCW4713).

^d^ Compared to the equivalent mean recombinant frequency for wild-type *RTS1-AO* with 5 kb DNA spacer (MCW8023).

^e^ Compared to the equivalent mean recombinant frequency for wild-type *RTS1-AO* with 2 kb spacer (MCW8020).

^f^ Data from Ref. (Osman et al., 2016)
