# Supplementary material for: Inter-Fork Strand Annealing causes genomic deletions during the termination of DNA replication
Source: eLife. 2017 Jun 6;6:e25490. doi: 10.7554/eLife.25490 (PMC5461108; doi:10.7554/eLife.25490)
Supplement: Supplementary file 1. — DOI: http://dx.doi.org/10.7554/eLife.25490.014 [file elife-25490-supp1.docx]

**SUPPLEMENTARY FILE 1**

*Schizosccharomyces pombe* strains

| **Strain** | **Relevant genotype** | **Source** |
| --- | --- | --- |
| MCW1452 | *h^?^ mus81*∆*::kanMX6 ade6-M375 int*::*pUC8/his3^+^/RTS1-AO/ade6-L469 ura4-D18 his3-D1 leu1-32 arg3-D4* | Lab strain |
| FO1742 | *h^+^ exo1*∆*::ura4^+^ ade6-M375 int*::*pUC8/his3^+^/RTS1-AO/ade6-L469 ura4-D18 his3-D1 leu1-32 arg3-D4* | Ref. (Osman et al., 2016) |
| MCW3061 | *h^+^ fml1*∆*::natMX4 ade6-M375 int*::*pUC8/his3^+^/RTS1-AO/ade6-L469 ura4-D18 his3-D1 leu1-32 arg3-D4* | Ref. (Sun et al., 2008) |
| MCW4712 | *h^+^ ade6-M375 int*::*pUC8/his3^+^/RTS1-IO/ade6-L469 ura4-D18 his3-D1 leu1-32 arg3-D4* | Lab strain |
| MCW4713 | *h^+^ ade6-M375 int*::*pUC8/his3^+^/RTS1-AO/ade6-L469 ura4-D18 his3-D1 leu1-32 arg3-D4* | Lab strain |
| MCW6395 | *h^+^ade6-M375 int*::*pUC8/lacO^115^/his3^+^/RTS1-IO/ade6-L469 lys1^-^*::*Pnmt41-NLS-lacI-tdKatushka2-hphMX4 rad52^+^*::*YFP-kanMX6 ura4-D18 his3-D1 leu1-32 arg3-D4* | Ref. (Nguyen et al., 2015) |
| MCW6556 | *h^+^ade6-M375 int*::*pUC8/lacO^115^/his3^+^/RTS1-AO/ade6-L469 lys1^-^*::*Pnmt41-NLS-lacI-tdKatushka2-hphMX4 rad52^+^*::*YFP-kanMX6 ura4-D18 his3-D1 leu1-32 arg3-D4* | Ref. (Nguyen et al., 2015) |
| MCW6778 | *h^+^oriIII-1253∆*::*natMX4 ade6-M375 int*::*pUC8/his3^+^/RTS1-AO/ade6-L469 ura4-D18 his3-D1 leu1-32 arg3-D4* | Ref. (Nguyen et al., 2015) |
| MCW7020 | *h^+^ ade6-M375 int*::*pUC8/his3^+^/RTS1-AO/500 bp spacer/ade6-L469 ura4-D18 his3-D1 leu1-32 arg3-D4* | this study |
| MCW7021 | *h^+^ ade6-M375 int*::*pUC8/his3^+^/RTS1-AO/1000 bp spacer/ade6-L469 ura4-D18 his3-D1 leu1-32 arg3-D4* | this study |
| MCW7022 | *h^+^ ade6-M375 int*::*pUC8/his3^+^/RTS1-AO/2000 bp spacer/ade6-L469 ura4-D18 his3-D1 leu1-32 arg3-D4* | this study |
| MCW7290 | *h^+^ Ter2/3-natMX4-aim1 ade6-M375 int*::*pUC8/his3^+^/RTS1-IO/ade6-L469 ura4-D18 his3-D1 leu1-32 arg3-D4* | this study |
| MCW7292 | *h^+^ Ter2/3-natMX4-aim1 ade6-M375 int*::*pUC8/his3^+^/RTS1-AO/ade6-L469 ura4-D18 his3-D1 leu1-32 arg3-D4* | this study |
| MCW7301 | *h^+^ Ter2/3-natMX4-aim1 ade6-M375 int*::*pUC8/lacO^115^/his3^+^/RTS1-IO/ade6-L469 lys1^-^*::*Pnmt41-NLS-lacI-tdKatushka2-hphMX4 rad52^+^*::*YFP-kanMX6 ura4-D18 his3-D1 leu1-32 arg3-D4* | this study |
| MCW7304 | *h^+^ Ter2/3-natMX4-aim1 ade6-M375 int*::*pUC8/lacO^115^/his3^+^/RTS1-AO/ade6-L469 lys1^-^*::*Pnmt41-NLS-lacI-tdKatushka2-hphMX4 rad52^+^*::*YFP-kanMX6 ura4-D18 his3-D1 leu1-32 arg3-D4* | this study |
| MCW7748 | *h^+^ ade6-M375 int*::*pUC8/his3^+^/RTS1-AO/8000 bp spacer/ade6-L469 ura4-D18 his3-D1 leu1-32 arg3-D4* | this study |
| MCW7749 | *h^+^ ade6-M375 int*::*pUC8/his3^+^/RTS1-AO/4000 bp spacer/ade6-L469 ura4-D18 his3-D1 leu1-32 arg3-D4* | this study |
| MCW8019 | *h^+^ ade6-M375 int*::*pUC8/1000 bp spacer/his3^+^/RTS1-AO/ade6-L469 ura4-D18 his3-D1 leu1-32 arg3-D4* | this study |
| MCW8020 | *h^+^ ade6-M375 int*::*pUC8/2000 bp spacer/his3^+^/RTS1-AO/ade6-L469 ura4-D18 his3-D1 leu1-32 arg3-D4* | this study |
| MCW8021 | *h^+^ ade6-M375 int*::*pUC8/3000 bp spacer/his3^+^/RTS1-AO/ade6-L469 ura4-D18 his3-D1 leu1-32 arg3-D4* | this study |
| MCW8022 | *h^+^ ade6-M375 int*::*pUC8/4000 bp spacer/his3^+^/RTS1-AO/ade6-L469 ura4-D18 his3-D1 leu1-32 arg3-D4* | this study |
| MCW8023 | *h^+^ ade6-M375 int*::*pUC8/5000 bp spacer/his3^+^/RTS1-AO/ade6-L469 ura4-D18 his3-D1 leu1-32 arg3-D4* | this study |
| MCW8134 | *h^+^ oriIII-1253∆*::*natMX4 ade6-M375 int*::*pUC8/5000 bp spacer/his3^+^/RTS1-AO/ade6-L469 ura4-D18 his3-D1 leu1-32 arg3-D4* | this study |
| MCW8136 | *h^+^ rad51*∆*::arg3^+^ ade6-M375 int*::*pUC8/5000 bp spacer/his3^+^/RTS1-AO/ade6-L469 ura4-D18 his3-D1 leu1-32 arg3-D4* | this study |
| MCW8138 | *h^+^ rad51*∆*::arg3^+^ rad52*∆*::ura4^+^ ade6-M375 int*::*pUC8/5000 bp spacer/his3^+^/RTS1-AO/ade6-L469 ura4-D18 his3-D1 leu1-32 arg3-D4* | this study |
| MCW8300 | *h^?^ fml1*∆*::natMX4 ade6-M375 int*::*pUC8/2000 bp spacer/his3^+^/RTS1-AO/ade6-L469 ura4-D18 his3-D1 leu1-32 arg3-D4* | this study |
| MCW8362 | *h^+^ ade6-M375 int*::*pUC8/5000 bp spacer/his3^+^/RTS1-IO/ade6-L469 ura4-D18 his3-D1 leu1-32 arg3-D4* | this study |
| MCW8402 | *h^+^ mus81*∆*::arg3^+^ ade6-M375 int*::*pUC8/5000 bp spacer/his3^+^/RTS1-AO/ade6-L469 ura4-D18 his3-D1 leu1-32 arg3-D4* | this study |
| MCW8434 | *h^+^ mus81*∆*::arg3^+^ ade6-M375 int*::*pUC8/2000 bp spacer/his3^+^/RTS1-AO/ade6-L469 ura4-D18 his3-D1 leu1-32 arg3-D4* | this study |
| MCW8456 | *h^+^ rad51*∆*::arg3^+^ oriIII-1253∆*::*natMX4 ade6-M375 int*::*pUC8/5000 bp spacer/his3^+^/RTS1-AO/ade6-L469 ura4-D18 his3-D1 leu1-32 arg3-D4* | this study |
| MCW8589 | *h^+^ exo1*∆*::kanMX6 ade6-M375 int*::*pUC8/5000 bp spacer/his3^+^/RTS1-AO/ade6-L469 ura4-D18 his3-D1 leu1-32 arg3-D4* | this study |
| MCW8592 | *h^?^ Ter2/3-natMX4-aim1 ade6-M375 int*::*pUC8/5000 bp spacer/his3^+^/RTS1-AO/ade6-L469 ura4-D18 his3-D1 leu1-32 arg3-D4* | this study |

Nguyen, M.O., Jalan, M., Morrow, C.A., Osman, F., and Whitby, M.C. (2015). Recombination occurs within minutes of replication blockage by *RTS1* producing restarted forks that are prone to collapse. Elife *4*, e04539.

Osman, F., Ahn, J.S., Lorenz, A., and Whitby, M.C. (2016). The RecQ DNA helicase Rqh1 constrains Exonuclease 1-dependent recombination at stalled replication forks. Sci Rep *6*, 22837.
