# Supplementary material for: Inter-Fork Strand Annealing causes genomic deletions during the termination of DNA replication
Source: eLife. 2017 Jun 6;6:e25490. doi: 10.7554/eLife.25490 (PMC5461108; doi:10.7554/eLife.25490)
Supplement: Supplementary file 2. — DOI: http://dx.doi.org/10.7554/eLife.25490.015 [file elife-25490-supp2.docx]

**SUPPLEMENTARY FILE 2**

Oligonucleotides

| Oligonucleotide | Nucleotide sequence (5´ to 3´) |
| --- | --- |
| oMW305 | GACGCTGCCGAATTCTACCAGTGCCTTGCTAGGACATCTTTGCCCACCTGCAGGTTCACCC |
| oMW306 | GGGTGAACCTGCAGGTGGGCGGCTGCTCATCGTAGGTTAGTTGGTAGAATTCGGCAGCGTC |
| oMW416 | TCATAGATATCCGGTCGATC |
| oMW417 | TATAGAACATCTTGCTCTTA |
| oMW421 | TAAGAGCAAGATGTTCTATAAAAGATGTCCTAGCAAGGCACGATCGACCGGATATCTATGA |
| oMW1556 | TTTTGTCGACGGTACCGAGCTCTAGAGGATCCGAATCCCCGTC |
| oMW1557 | TATAGTCGACTAGTCCCGGGATCCAAGGAATTGGTAGTG |
| oMW1558 | TTGGTACCACTAGTTCCGACAGCATCGCCAGTC |
| oMW1560 | TATACCCGGGAGCTCATAAGTGCGGCGACGATAG |
| oMW1561 | TATACCCGGGAGCTCTCCGCAAGAATTGATTGG |
| oMW1562 | TATACCCGGGAGCTCGTGCTACGCCTGAATAAGTG |
| oMW1643 | TTGGTACCACTAGTAACCTGTATCAGAACATGC |
| oMW1644 | TACCCGGGAGCTCAGGACGATATTGTAAATTCAC |
| oMW1700 | TATACAGCTGGCGCGCCCAATGCGCTTACTGATGC |
| oMW1701 | TATACAGCTGCTAGCCACCGGAGAAACTAACGAC |
| oMW1712 | TATAGCTAGCGTCGCTGTCTCGCCACACGTC |
| oMW1713 | TATAGCTAGCCAGTCTGCGTGCTACTTCTGTCTG |
| oMW1714 | TATAGCTAGCGGCTGAGTTCGTGCTTACCGCAG |
| oMW1715 | TATAGCTAGCATGCACGGGCAAAACGGCAGGAG |
